# Elucidating Structural Disorder in a Polymeric Layered Material: The Case of Sodium Poly(heptazine imide) Photocatalyst

**DOI:** 10.1021/acs.nanolett.5c04946

**Published:** 2025-12-01

**Authors:** Daniel Khaykelson, Gabriel A. A. Diab, Sidney R. Cohen, Tamar Kashti, Tatyana Bendikov, Iddo Pinkas, Ivo F. Teixeira, Nadezda V. Tarakina, Lothar Houben, Boris Rybtchinski

**Affiliations:** † Department of Molecular Chemistry and Materials Science, 34976 Weizmann Institute of Science, Rehovot 7610001, Israel; ‡ Department of Chemistry, Federal University of São Carlos, São Carlos, São Paulo 13565-905, Brazil; § Department of Chemical Research Support, Weizmann Institute of Science, Rehovot 7610001, Israel; ∥ AI Hub, The Institute for Artificial Intelligence, Weizmann Institute of Science, Rehovot 7610001, Israel; ⊥ Department of Colloid Chemistry, Max Planck Institute of Colloids and Interfaces, Research Campus Golm, Am Mühlenberg 1, 14476 Potsdam, Germany; # INM-Leibniz Institute for New Materials, Campus D2 2, 66123 Saarbrücken, Germany; ## Department for Materials Science and Engineering, Saarland University, 66123 Saarbrücken, Germany

**Keywords:** disordered materials, 2D materials, mesoscale, 4D-STEM, machine learning, semi-crystalline materials

## Abstract

Structurally heterogeneous materials present major challenges for characterization due to their complex nanoscale order. Sodium poly­(heptazine imide) (NaPHI), a layered carbon nitride photocatalyst, exemplifies this complexity, with its precise structure remaining unresolved. Here, we uncover new structural insights into NaPHI using energy-filtered four-dimensional scanning transmission electron microscopy combined with machine-learning-based diffraction image segmentation, supported by transmission electron microscopy, atomic force microscopy, X-ray diffraction, and Raman spectroscopy. At the mesoscale, NaPHI flakes display bent morphologies, while nanodiffraction patterns reveal features characteristic of stacking disorder. Based on these insights, we modeled a NaPHI-layered structure incorporating out-of-plane undulations (waves) with amplitudes of ∼0.5 Å and wavelengths of 2–3 nm. This model reproduces the observed line features in nanodiffraction patterns and agrees with powder X-ray diffraction data, thereby bridging local and bulk structural information. The introduced approach uses data-driven machine learning to identify statistically significant features, offering a robust framework for structural analysis of semi-crystalline materials.

## Graphical abstract

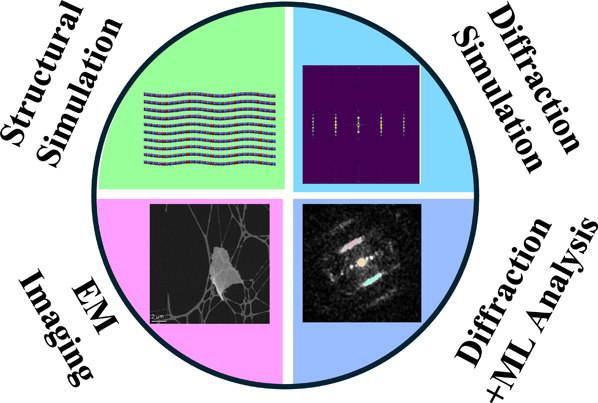

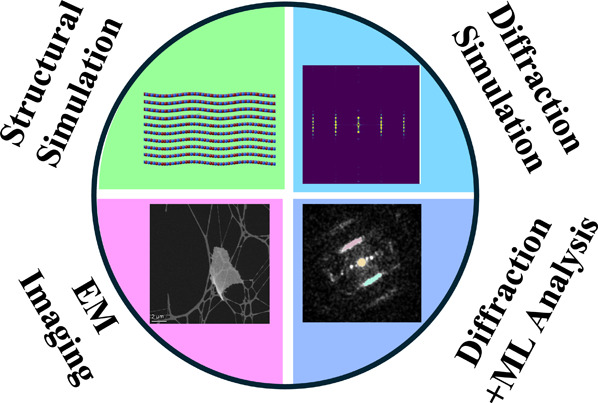

Crystalline functional materials often exhibit structural defects and inhomogeneities, such as polycrystallinity, lattice strain, crystal bending, and other forms of structural disorder.^1–5^ While bulk crystal structures can be inferred using methods such as powder X-ray diffraction (pXRD), obtaining insight into nanoscale structural heterogeneity remains challenging. This complexity is pronounced in 2D layered crystalline materials due to the contrast between their weak interlayer and strong intralayer bonding, which makes them susceptible to structural fluctuations.^6,7^ Graphitic carbon nitrides are one such family that has recently attracted significant attention as transition-metal-free photocatalysts.^8–11^ Specifically, poly­(heptazine imides) (PHIs) have been extensively studied for this application due to their advantageous optoelectronic properties and straightforward synthesis.^12,13^ PHIs consist of 2D polymeric networks of heptazine (tri-*s*-triazine) units interconnected by negatively charged nitrogen bridges, which are counterbalanced by alkali metal cations. Their layered structure is stabilized by π–π stacking interactions. Variations in synthesis conditions can significantly affect crystallinity and defect types, enabling the coexistence of multiple structural polytypes.^10,14^ Additionally, the amount and type of metal incorporated into the PHI framework influence heptazine layer stacking and induce local structural changes, thereby affecting interlayer charge transfer and photocatalytic activity.^8,10,13,15^ Analyzing local structural disorder in PHIs remains a significant challenge.^1^

In this work, we present a methodology that bridges bulk and nanoscale structural analysis in semi-crystalline materials by integrating electron nanodiffraction, machine learning, and simulations to resolve the mesoscale structure. Applied to PHIs, the approach uncovers structural inhomogeneities and periodic disorder, enabling structure elucidation. More broadly, this framework addresses a critical gap in structural science and is applicable to a wide range of semi-crystalline materials.

NaPHI flakes were prepared using a previously published procedure^16^ based on a bottom-up ionothermal pyrolysis of a N-rich precursor (melamine) in the presence of a structure-directing salt, NaCl. Powder X-ray diffraction (pXRD), Raman spectroscopy, X-ray photoelectron spectroscopy (XPS), and X-ray energy-dispersive and electron energy loss spectroscopies (EDS and EELS, respectively) were employed to characterize the material, confirming NaPHI formation (see the Supporting Information for details). High-resolution transmission electron microscopy (HR-TEM) images of NaPHI revealed a dominant spacing of 1.1 nm, corresponding to the *a* lattice parameter in the hexagonal unit cell,^1,10,15,17^ and structural heterogeneity, such as multi-layer multi-angle rotational domains reported previously (Figure 1c and Figure S1).^1^ The presence of many off-axis domains and the lack of in-plane domains in the field of view in Figure 1c (see also Figure S1) are consistent with the bent nature of NaPHI flakes.

**Figure 1:**
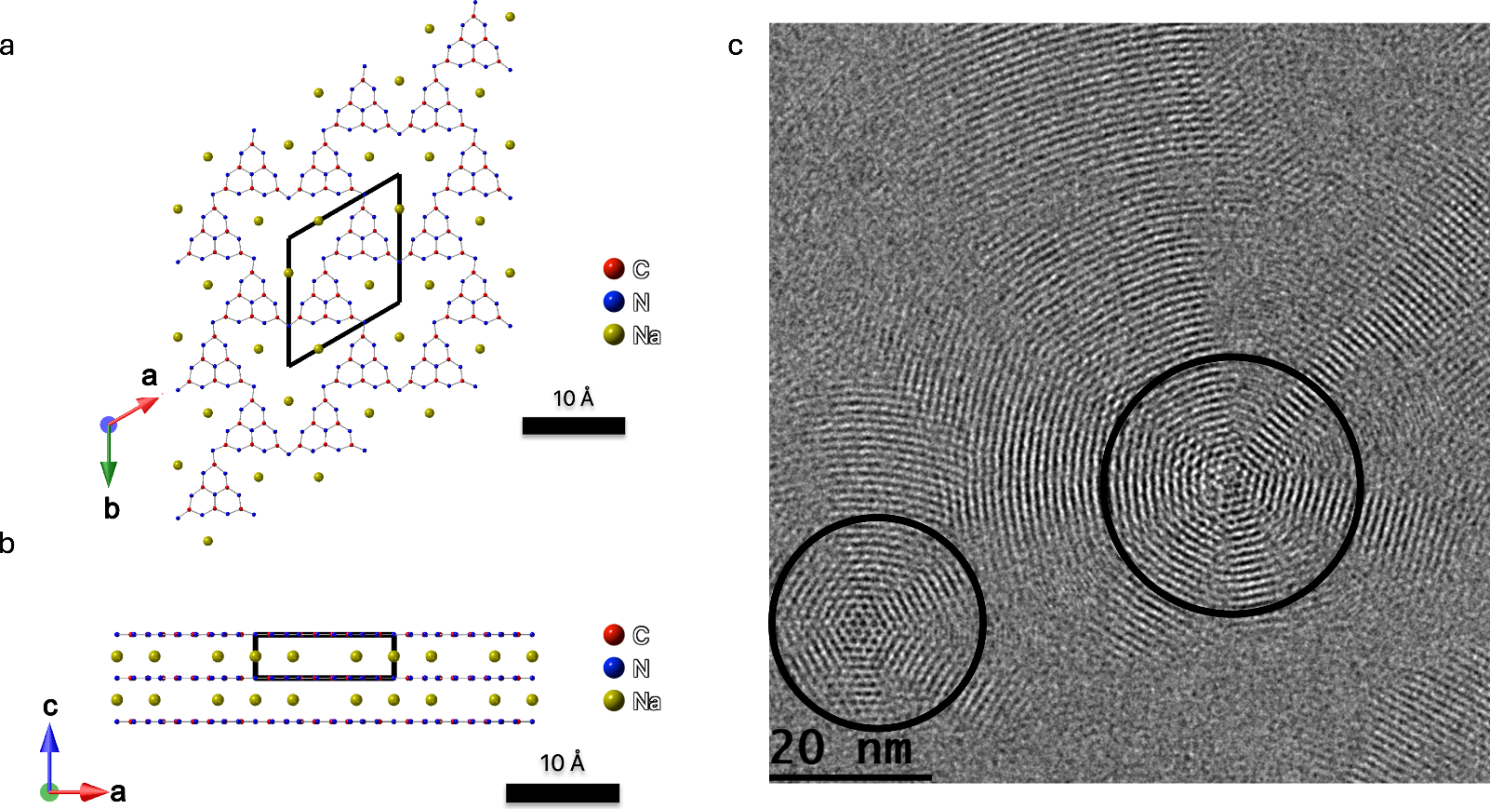
(a) Projection along the *c* axis of NaPHI,^1^ showing the (001) basal plane. (b) Projection along the *b* axis of NaPHI,^1^ showing its stacking nature. (c) HR-TEM image of NaPHI. Two multi-angle rotational domains are marked by black circles. Non-crystalline areas can be attributed to off-focus domains due to bending.

STEM tilt series and AFM were employed to further investigate the NaPHI morphology (Figure 2, Figure S2, and Supporting Movies 1 and 2). The analysis revealed the presence of layered aggregates of various sizes, consistent with the “layered islands” growth mechanism suggested for PHIs.^18–21^ In Figure 2, we present an example of a micrometer-sized concave flake, where a tilt of 45° revealed its curvature. AFM images (Figure 2 and Figure S2) were used to determine the apparent curvature radius. A dozen independent measurements on four different flakes gave curvature radii ranging from 380 nm up to 4.5 μm with a corresponding average of 2.3 μm and standard deviation of 1.9 μm. These results further support the link between heterogeneity revealed in the TEM measurements and bulk structure.

**Figure 2:**
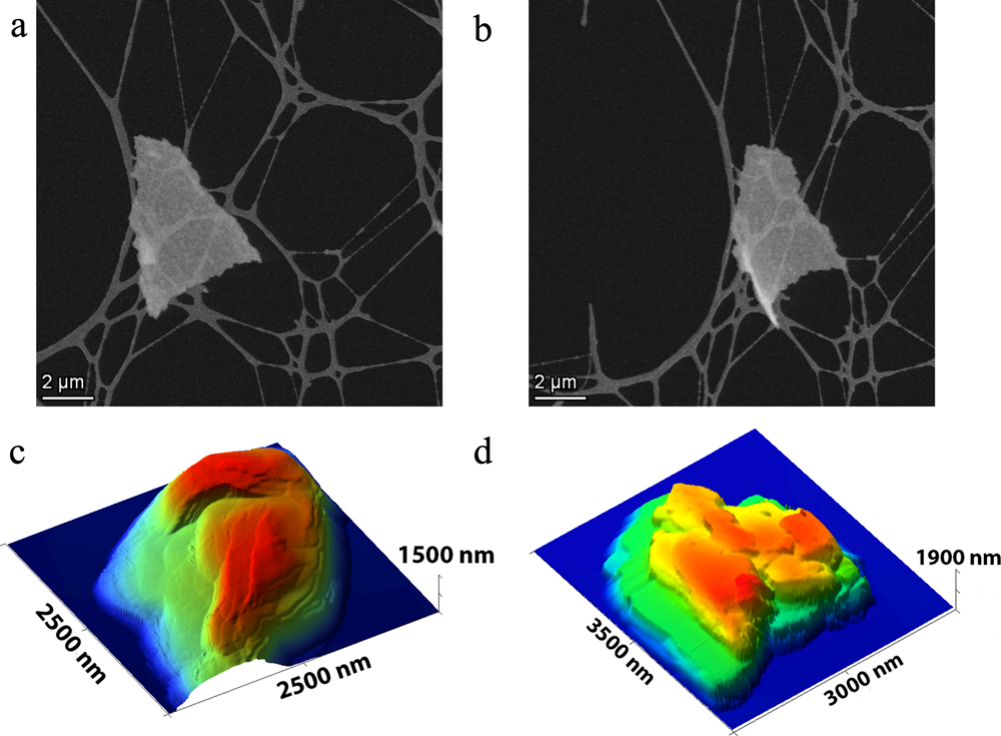
Bending in NaPHI films. (a and b) Single STEM frames from STEM tilt series, at 0° and +45° angles, respectively. (c and d) AFM images of NaPHI, revealing curvature and multi-layered structure.

Energy-filtered four-dimensional scanning transmission electron microscopy (4D-STEM) was employed to investigate the crystallinity of NaPHI in greater detail (Figures 3 and 4). Measurements were conducted under low-dose conditions using direct electron detection in an energy-filtered setup with a total fluence of 50 e^–^/Å^2^, as required due to the beam-sensitive nature of PHIs. Crystalline order appears as sharp spots in the diffraction patterns, while structural disorder gives rise to elastic diffuse scattering features, such as halos, arcs, lines, and speckles.^22–24^ Identifying the structural origins of such features is particularly challenging in complex materials and almost impossible using “bulk” diffraction methods.

**Figure 3:**
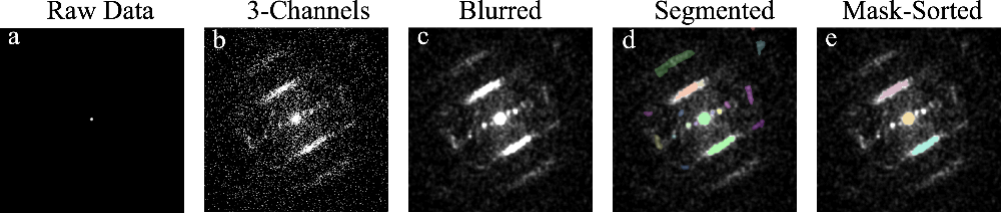
Meta’s Segment Anything Model (SAM) application example. (a) Diffraction of raw images without a beam stop usually appears as the central beam, as diffraction is a rare event. (b) matplotlib’s histogram stretching of the image and transforming the gray image to RGB for SAM input. (c) After Gaussian blur of size 33 × 33 pixels (σ = 4) due to low-dose conditions (∼50 e^–^/Å^2^), the images are noisy and some lines are not continuous. (d) Example of the output of SAM. In this example, SAM detected 17 masks, each with associated box parameters (position, side lengths, etc.) used for filtering. (e) Chosen masks based on the box aspect ratio, size, length, and position to fit the features of interest. From the 17 detected, three specific masks were selected, highlighted by their distinct colors. The central mask (yellow) was chosen as the one closest to the center of the image, while the line masks (pink and cyan) were selected based on their aspect ratio and *q* distance.

**Figure 4:**
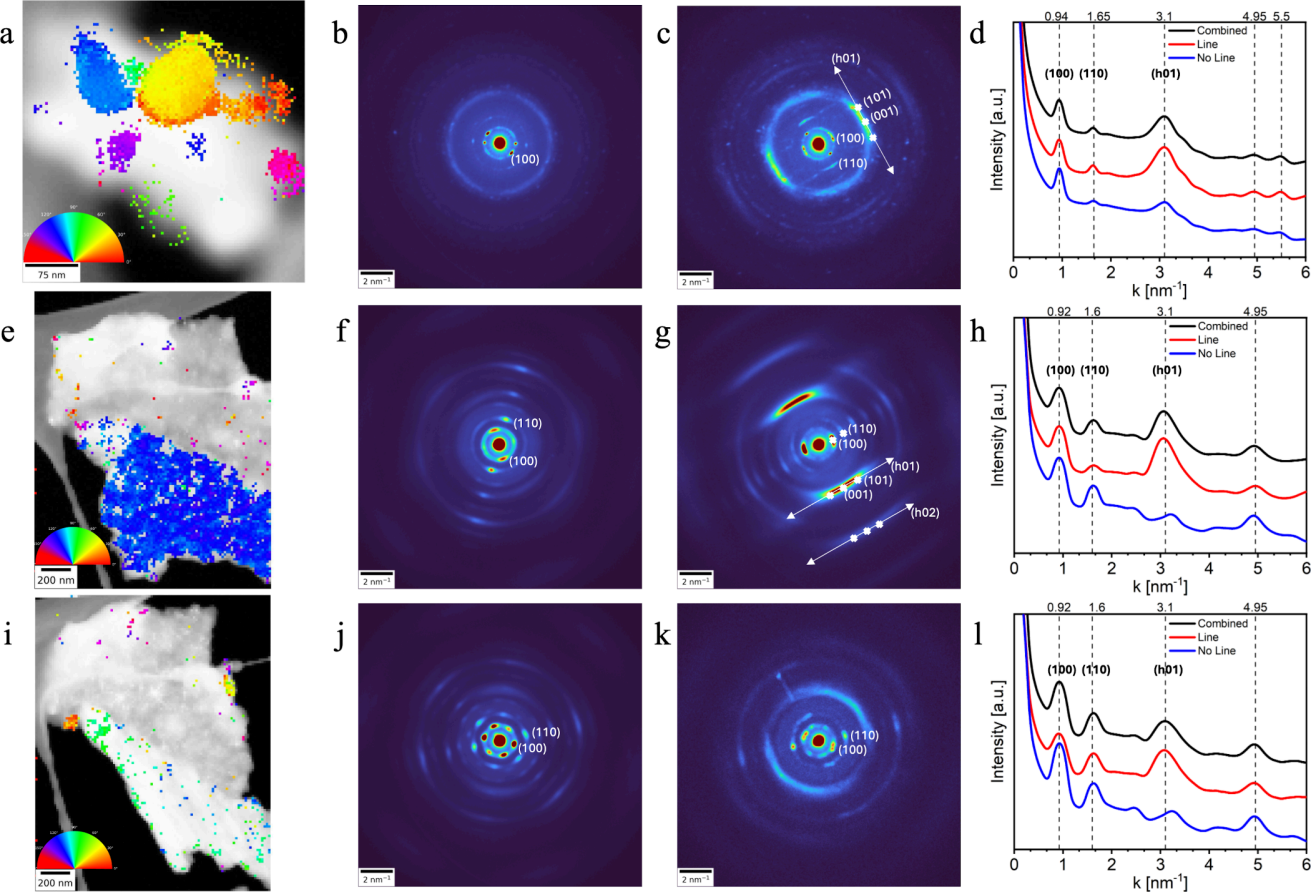
4D-STEM of NaPHI. (a, e, and i) Virtual bright-field and line-orientation images of (a) a small NaPHI flake and a mature flake viewed from (e) 0° and (i) 45° tilts, respectively. Colored regions correspond to line-like diffraction around the 3 Å spacing (001). The line features are tilt-dependent, as they are much less abundant at 45° tilt. (b, f, and j) Average diffraction patterns from the line-free (colorless) region in panels a, e, and i, respectively. (c, g, and k) Average diffractions from colored regions in panels a, e, and i, respectively. White x markings in panels c and g are the simulated position of peaks, and the white line is used to guide the eye. Tilting from 0° to 45° decreased the amount of line-like diffraction patterns around the *c* vector and increased the in-plane *a* = *b* vector diffraction, indicating their orientational dependence. (d, h, and l) Radially averaged plots from the colorless areas (line-free), colored areas (line features), and a combination of both for panels a, e, and i, respectively. Peaks at *d* = 0.92/0.94, 1.6, and 3.1 nm^–1^ are correlated to the (100)/(010), (110), and (001) planes, respectively.

In order to probe the complex nature of structural disorder within the NaPHI lattice, we applied energy-filtered 4D-STEM combined with a diffraction classification approach based on machine learning (ML) using the Segment Anything Model (SAM) by Meta (Figure 3).^25^ Unlike conventional methods, such as blob analysis or Hough/Radon transforms,^26^ which require prior assumptions about the featured geometry (e.g., disks or lines), its size, and relative intensity, SAM operates without any initial structural bias, providing an unsupervised feature extraction. It segments prominent diffraction features (e.g., spots, central beam, streaks, and diffuse lines) from the background without the need for predefined templates. The only required pre-processing is replicating the single channel (Figure 3a) into a three-channel image (Figure 3b) to satisfy SAM’s input. This operation preserves pixel values; any apparent difference between Figure 3a (one channel) and Figure 3b (three channels) is due solely to matplotlib’s^23^ rendering defaults; grayscale images are auto-normalized and color-mapped, whereas RGB images are displayed without normalization. Because the data were acquired at a relatively low dose (∼50 e^–^/Å^2^), we applied a mild Gaussian blur before segmentation to suppress noise and prevent the line feature from fragmenting (appearing non-continuous) in some frames (Figure 3c; code is available in Github^27^). The resulting segments were converted into masks to spatially localize their origins within the material (Figure 3d). These were then categorized by the physical property of interest, for example, the central beam by selecting the mask nearest the image center and line features by selecting masks with a high aspect ratio (Figure 3e). This reframes feature extraction from iterative parameter tuning of computer-vision methods to domain-expert curation of the segmentation outputs. Overall, the SAM-based workflow enabled robust identification of both crystalline and diffuse scattering features, offering a powerful tool for processing information on structural disorder present in reciprocal space, which is essential for the purpose of modeling and quantitative description.

The classification using SAM reveals an intricate multidomain architecture (Figure 4), resulting in the orientation-dependent diffraction behavior of NaPHI that was analyzed by using line-feature mapping. Among the various features, the line patterns (Figure 4c and g) emerged as dominant across multiple NaPHI diffraction frames, suggesting their central role in the observed deviations from ideal crystallinity. The key finding is that diffuse lines connect the systematic rows of Bragg reflections (*h*01) with a non-vanishing component *h* along the layer stacking direction. They are absent in the central row of (*h*00) reflections, which means that the diffuse lines are not to be confused with streaks originating from shape effects. The diffuse lines along the (*h*01) rows are highly sensitive to sample orientation, disappearing upon tilting. Figure 4a–d shows 4D-STEM data from a small, less mature NaPHI aggregate. Line-feature segmentation using Meta SAM revealed multiple domains with a rotational offset (colored areas in Figure 4a), while colorless regions lacked these line features. The corresponding averaged diffraction patterns from line-free and line-rich areas are shown in Figure 4b and c, respectively. The line-free regions (Figure 4b) exhibit diffuse halos, indicating a rotational disorder. In contrast, the line-rich regions (Figure 4c) show distinct diffraction lines that correspond to an interlayer reciprocal distance of ∼3.1 nm^–1^ along the *c* axis. Radial profiles (Figure 4d) reveal three broad peaks at 0.94, 1.65, and 3.1 nm^–1^, corresponding to the (100), (110), and (001) planes, respectively. These peaks confirm that the internal orientation of the crystallites in smaller aggregates is highly tilted from an in-plane configuration. Due to the equivalence of the [100] and [010] viewing directions, (*h*01) can be symmetrically equivalent to (0*k*1).

To investigate the orientational dependence of the line features, a large NaPHI flake was imaged at 0° (Figure 4e–h) and 45° (Figure 4i–l) tilts. At 0°, the diffraction is dominated by off-axis line features corresponding to a single domain (blue, Figure 4g). Diffraction from the colorless (line-free) areas (Figure 4f) contains a central halo, a weak central line, and smeared hexagonal features at higher angles, consistent with a mix of tilted and in-plane domains. Radial profiles (Figure 4h) confirm the presence of (100) and (110) peaks from the central line, while the broad (001) peak is mainly restricted to the line-rich (red) regions. Upon tilting to 45° (Figure 4i–l), the line features vanish (Figure 4i), being replaced by a strong 6-fold diffraction pattern (Figure 4j), characteristic of in-plane alignment. The few remaining line-containing regions (Figure 4k) exhibit less homogeneity, but their radial profiles (Figure 4l) closely resemble those at 0° (Figure 4h) and the non-mature aggregate (Figure 4d). This suggests that line features reflect high-tilt orientations and highlight the value of angular diffraction data for probing the local structure. Diffraction line features in NaPHI are strongly orientation-dependent and always contain an out-of-plane component of the *ab* plane, which vanishes in-plane, while in-plane domains give rise to distinct 6-fold patterns upon tilting. The spatially resolved diffraction data of NaPHI indicate that its layered structure is intrinsically disordered, giving rise to non-trivial diffraction line patterns perpendicular to the (*h*00) diffraction plane. We hypothesized that the smearing of diffraction into lines at higher angles, while having well-defined diffraction spots in the central rows, arises from a non-trivial deformation in the stacking plane. To verify our hypothesis, we simulated NaPHI monolayers arranged into atomistic supercells, and the resulting models were used to calculate the 2D diffraction patterns, employing multi-slice simulation with the abTEM package.^28^ The ions were previously reported to be disordered within the NaPHI crystalline backbone,^1^ which was also tested in our models. Various forms of stacking disorder were implemented in the model, and the layer stacking disorder was iteratively refined after comparing the calculations to the experiment. NaPHI monolayers were first stacked in an eclipsed way, using a previously published model of NaPHI,^1^ including various stacking faults, which did not successfully reproduce the diffuse scattering (Figures S3–S5). A good match between the theory and experiment was obtained with a NaPHI array having a wave-like deformation propagating perpendicular to the *c* axis (perpendicular to the stacking direction; Figure 5a). This buckling model reproduced the observed structural pattern: sharp diffraction spots on the central axis, and lines perpendicular at the ∼3.1 nm^–1^ distance (and higher harmonics; Figure 5b), when viewed from higher angles (90°). The presence of the ions did not cause the line features in the structures without wave deformations (Figure S6), while they were included in models containing waves, reproducing the line features (Figure 5 and Figure S7).

**Figure 5:**
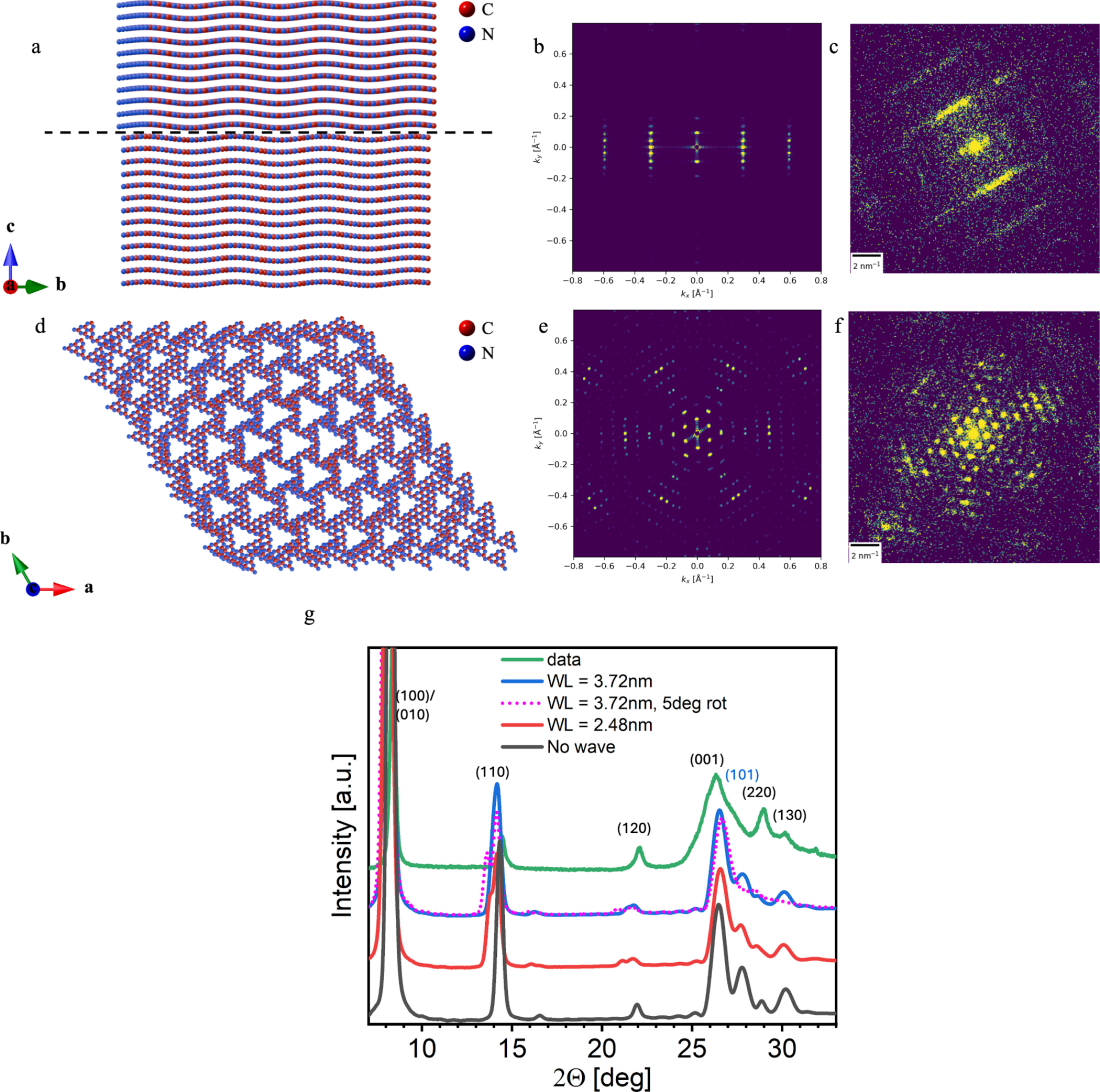
Structural simulation of deformations in NaPHI generated from a previously published model.^1^ (a) Representative view of NaPHI viewed from the [100] direction (perpendicular to the *c* axis). The wave amplitude is 0.5 Å, and the wavelength is 24.8 Å (unit cell × 2). The supercell is 8 × 8 × 8 nm in size. Supercell parameters were chosen to be close to the STEM probe size. They show a single moiré pattern and reduce central line periodicity (affected by larger *ab*). The rotation angle is 5° between two stacks of layers (the dashed line represents the rotational interface). Na^+^ ions (approximately 1.5 ions per channel on average^1^) are not shown to simplify viewing (full structure in Figure S7). (b) Simulated diffraction of the structure from the model in panel a. Interlayer stacking is modulated due to the wavy structure. The larger in-plane (channel) *d* ∼ 1 nm spacing appears as a sharp central peak. The smearing of the stacking peaks while maintaining sharp peaks in the central line is a result of the wave being perpendicular to the stacking (*c* axis) direction. (c) Single frame from NaPHI showing similarity to the simulation. (d) Same structure as in panel a but viewed from the top (*c* axis) [001] direction (rotated 90° relative to panel a). A single moiré pattern of approximately 8 nm is visible. (e) Simulation from panel d. (f) Single frame shows diffraction similar to the one in panel e. (g) pXRD data (green) and simulations of flat (no waves, black), wavy NaPHI with two wavelengths: 24.8 Å (twice the unit cell, red) and 37.2 Å (3 times the unit cell, blue) and 37.2 Å wavelength with rotation domain (pink, dots) showing broadening and smearing of the 001/101 peaks.

We further tested several parameters related to the model. Figure 5c shows a single experimental diffraction frame and its similarity to the simulated NaPHI signal in Figure 5b. Various wavelengths and amplitudes were tested (Figure 5), and the smallest simulated wave amplitude and wavelength are ∼0.5 and ∼24.8 Å (2 times the in-plane unit cell), respectively. The in-plane rotational diffraction was analyzed by tilting the same model to an in-plane orientation and introducing a 5° rotation between two sets of stacked monolayers (Figure 5d). The simulated diffraction (Figure 5e) is very similar to the experimental one (Figure 5f). The simulated model included both waves and rotational domains, emphasizing the separation of the line features and in-plane diffraction. We note that the rotational moiré pattern (periodicity of approximately 8 nm; Figure 5d) is similar in appearance to the one observed in the TEM image (about 10 nm; Figure 1). It is important to note that the moiré patterns differ across the TEM images (Figure 1c and Figure S1), indicating variability in the rotation angles. With regard to the variability of the wave pattern, the structure retains its characteristic line features in the simulated diffraction images when up to 10% randomness is introduced in the wavelength and amplitude of the waves (each wave is perturbed by a random factor between 0 and 10%; Figure S8). At higher degrees of randomness, the diffraction pattern is no longer reproducible.

We could further relate the disorder in NaPHI to the (001) broadening feature, as observed in the powder X-ray diffraction (Figure 5g). Such broadening has been reported for 2D materials and was explained with a turbostratic stacking disorder,^29^ referring to some degree of general rotation or slip of the 2D lattice planes relative to each other. When comparing NaPHI pXRD patterns to other PHIs, the (001) peak is often asymmetrically broadened, having satellite peaks.^1,30,31^ Apparently, the anisotropic nature of the broadening is related to the line features: unlike in halos, points on straight lines do not have the same distance from the center, leading to a large-angle tail of the peak. To prove that this is the case for the buckling disorder, pXRD patterns were simulated from our model structures (Figure 5g) using the Debye scattering equation.^32^ The similarity to the experimental data is evident, with broadening of the (*h*01) peak and shift of the (100)/(010) peak due to layer bending.^11^ To account for the possible polydispersity in wave features (Figure S9), two periodicities were simulated: one with a 24.8 Å wavelength (twice the unit cell; Figure 5g, red) and one with a 37.2 Å wavelength (thrice the unit cell; Figure 5g, blue), corresponding to three unit cells. Both simulations had a 0.5 Å amplitude. Rotation was introduced to the 24.8 Å wavelength model by rotating half of the layered stacks 5° relative to the other stack, as presented in Figure 5a and d. The rotation broadened the anisotropic peak, smoothing it and considerably reducing the (110) peak intensity (Figure 5g, pink, dashed). The broadening and smearing of the peak at 28° are apparent. The effect of the rotation in such a model is amplified relative to a real pXRD sample, as the rotational domains appear in only a fraction of the material. Overall, NaPHI gives rise to a characteristic structural model type, but its heterogeneity introduces intrinsic variance within the model, as expected for structurally disordered materials.

Our methodology enabled the elucidation of structural heterogeneities and periodic deformations in NaPHI. While local diffraction data show a clear difference between signals, the averaged diffraction data lack sensitivity to differentiate various structural features, necessitating the analysis of spatially resolved diffraction data in two or more dimensions that 4D-STEM can provide. The main disorder features in the two-dimensional diffraction of NaPHI are diffuse lines, qualitatively observed in single frames and independently evidenced with statistical significance by unsupervised ML, and layer diffraction spots attributed to the (*h*00) [=(0*k*0)] and (110) planes. Modeling and simulation revealed that the diffuse scattering in the diffraction patterns of NaPHI arises from wave-like deformations in the *ab* plane (perpendicular to the stacking direction), with wavelengths larger than 1.5–2 times the unit cell (1.86–2.48 nm; Figure 4 and Figure S9). In-plane orientational domains were also present and could be simulated with rotation angles up to 10°. The previously reported characteristics of pXRD spectra with an asymmetric (*h*01) peak broadening could be reproduced based on our model refinement and attributed to the wave-like deformation. Diffraction line features were previously observed in 2D covalent organic frameworks (COFs)^33^ and NaPHI^34^ but were not explicitly related to specific structural features. For PHIs, wave-like structural distortions were proposed from DFT modeling, with experimental diffraction data consistent with, but not directly resolving these features.^11,35^ Here, we directly correlate such diffraction features with structural modulations in PHI for the first time.

In summary, combining 4D-STEM, machine-learning-assisted diffraction pattern classification, and structural simulations enabled us to elucidate the structure of stacked NaPHI 2D crystals exhibiting wave-like deformations. Using segmentation-assisted mapping, we extracted subtle scattering features from noisy, diffuse-dominated patterns, allowing mapping of hidden structural motifs in NaPHI. Integrating 4D-STEM, ML diffraction analysis with forward simulations, we demonstrate a broadly applicable methodology for resolving nano- and mesoscale order inhomogeneities. Such a workflow facilitates identifying and uncovering hidden features, such as wave-like distortions and angular misalignments, which are key to understanding complex stacking behavior while minimizing user bias and enhancing feature discovery. Unlike traditional iterative refinement techniques, our data-driven approach uses machine learning to identify statistically significant features, offering a robust framework for structural analysis of semi-crystalline materials.

## Supplementary Material







## Notes

Detailed experimental methods and supplementary results, including 13 figures and a detailed description of the Segment Anything Method (PDF)
Supporting movie 1 of the STEM tilt series (MPG)
Supporting movie 2 of the STEM tilt series (MPG)
