# Supplementary material for: Elucidating Structural Disorder in a Polymeric Layered Material: The Case of Sodium Poly(heptazine imide) Photocatalyst
Source: Nano Lett. 2025 Dec 1;25(49):17230–6. doi: 10.1021/acs.nanolett.5c04946 (PMC12874626; doi:10.1021/acs.nanolett.5c04946)
Supplement: Supplementary file 1 [file nl5c04946_si_001.pdf]

Supporting Information

Elucidating Structural Disorder in a Polymeric Layered Material: The Case of Sodium-(Poly)-heptazine Imide Photocatalyst

Daniel Khaykelson, [a]* *Gabriel A. A. Diab,* [b] *Sidney R. Cohen,* [c] *Tamar Kashti,* [d] *Tatyana Bendikov,* [c] *Iddo Pinkas,* [c] *Ivo F. Teixeira,* [b] *Nadezda V. Tarakina,* [e,f] *Lothar Houben,* [c]* *Boris Rybtchinski* [a]*

[a] Department of Molecular Chemistry and Materials Science, Weizmann Institute of Science, Rehovot 7610001, Israel

[b] Department of Chemistry, Federal University of São Carlos, 13565-905, São Carlos, SP, Brazil

[c] Department of Chemical Research Support, Weizmann Institute of Science, Rehovot 7610001, Israel

[d] AI Hub, The Institute for Artificial Intelligence, Weizmann Institute of Science, Rehovot 7610001, Israel

[e] Department of Colloid Chemistry, Max Planck Institute of Colloids and Interfaces, Research Campus Golm, Am Mühlenberg 1, 14476 Potsdam, Germany

[f] INM - Leibniz Institute for New Materials and Saarland University, Campus D2 2, Saarbruecken, Saarland 66123, Germany

* daniel.kh@weizmann.ac.il; lothar.houben@weizmann.ac.il; boris.rybtchinski@weizmann.ac.il

Experimental Section

NaPHI synthesis: NaPHI was synthesized according to previously published procedures[1]. In short, melamine and NaCl were mixed via ball milling, heated to 600°C under nitrogen flow, and then cooled down. NaCl was washed using deionized water and the material was dried overnight at 60°C.

AFM: Atomic force microscopy (AFM) was performed on a modified Smart AFM (Horiba) in AC mode, using an 240AC-NA probe (NanoAndMore GMBH). Image analysis was performed using Gwyddion Open Source sofware.[2]

Electron Microscopy: Electron microscopy data were collected on a double aberration-corrected Themis-Z microscope (Thermo Fisher Scientific Electron Microscopy Solutions, Hillsboro, USA) equipped with a high-brightness FEG at an accelerating voltage of 200 kV. HR-TEM images were recorded on a Gatan OneView CMOS camera (Gatan Inc., Pleasanton, USA).
STEM images were obtained with a Fischione Model 3000 HAADF detector (E.A. Fischione Instruments, Export, PA). The inner collection cut-off angle was set to 60 mrad. EDS hyperspectral data were obtained with a Super-X SDD detector and quantified with the Velox software (TFS) through background subtraction and spectrum deconvolution.
Zero-loss filtered 4D-STEM data were collected on a DECTRIS ELA (DECTRIS AG, Baden, Switzerland) hybrid-pixel array detector, mounted on a CEOS CEFID (CEOS GmbH, Heidelberg, Germany) post-column energy filter. The energy-selecting slit of 10 eV was centered around the zero-loss peak. The electron pobe size containing 50% of electrons was approximately 6 nm at a convergence semi-angle of 0.2 mrad. A 3-4 pA beam-current and a 5 ms exposure time resulted in less than 50 $e^-/Å^2$ per exposure. Real-space sampling step-size was larger than the probe size, 8 nm for Figure3,A and 20 nm for Figure 3, E,I, for example, to avoid radiation damage from dose cumulation by beam overlap.
Electron energy loss spectra were measured with the CEOS CEFID spectrometer in STEM mode at a beam current of 200 pA, a semi-convergence angle of 21 mrad, and a semi-collection angle of 60 mrad using the ELA direct detection camera.

XPS: XPS measurements were carried out with Kratos AXIS ULTRA system using a monochromatic Al Kα X-ray source (hν = 1486.6 eV) at 75W and detection pass energies ranging between 20 and 80 eV. Low-energy electron flood gun (eFG) was applied for charge neutralization. Curve fitting analysis was based on linear or Shirley background subtraction and application of Gaussian-Lorenzian line shapes.

Raman Spectroscopy: Raman scattering measurements in the range of 70—3200 cm^{-1} were collected in the backscattering mode using a LabRAM HR Evolution (Horiba, France) confocal microspectrometer. Laser at 633 nm was used. The maximum incident power on the sample was 1–2 mW with submicron spot size. The Raman spectra were collected by a 1024 × 256-pixel open electrode front-illuminated CCD camera (Syncerity, Horiba, USA) cooled to −60 °C. The spectra reported are averages of laser exposure times of 20–50 s to achieve the best signal-to-noise ratio (SNR). The spectra were baseline corrected.

Computational Section

Segment-Anything Analysis: Meta's Segment Anything (SAM)[3] was used off-the-shelf without fine-tuning, employing the ViT-Huge model. Raw 4D-STEM data were uploaded as a .prz file, and each raw image was extracted without metadata. Images were originally grayscale and were transformed to a 3-channel representation to work with SAM via OpenCV[4], which also automatically rescaled viewing intensity, but does not change the data, when using matplotlib.pyplot[5] (Figure 3a-b). A blurring step was performed to reduce noise, followed by image resizing, both using the scikit-image library.[6] Each image was analyzed via SAM. Specific masks of interest were selected by filtering based on shape and position parameters of the mask boxes, using criteria such as:

- **Mask area range**, to exclude very small or very large masks;
- **Aspect ratio threshold**, to select elongated features by requiring masks to exceed a minimum aspect ratio;

- **Minimum line length**, to reject small blobs and prioritize extended line features;
- **Minimum and maximum distance from the image center**, to remove central beam artifacts and distant noise;
- **Minimum linear fit R^2**, to ensure masks correspond to well-defined linear features.

An example of image outputs at each processing step is detailed in Figure 3. All calculations were performed on a single NVIDIA RTX4080 GPU. Two false negatives were observed during analysis: (1) overly strong blurring merged line features with central-beam noise into a single large disk-like mask, and (2) insufficient blurring caused line features to fragment into small patches, leading to missed detections during sorting. Suggestions for addressing these false negatives are included in the code but were not applied in this work, as they affect only the size—not the nature—of the line domains. All code is available as Python notebooks at the Git repository[7].

Structural and Diffraction Simulations: Structural simulations were done using the Atomic Simulation Environment (ASE)[8]. Diffraction simulations were done using abTEM[9]. A monolayer of NaPHI[10] was extracted via ASE, and then geometric wave modifications were applied. For rotations, the final structure was divided to two parts, with one rotated by a certain degree relative to the other. The rotation interface is ephashized in figure 5a by a dashed line. The resulting structures were input to abTEM for electron-diffraction simulations. All code is available at the Git[11]. XRD powder patterns were calculated by solving the Debye scattering[12] equation, which predicts the scattered intensity in powder diffraction patterns to first order for gases, liquids, and randomly distributed nanoclusters in the solid state. A custom-written program[13] allowed us to calculate the diffraction pattern for a specific supercell, which is not periodic. Summation over a gallery of different supercells, which represent the possible configurations, provided a representative diffractogram for the full gallery of cases.

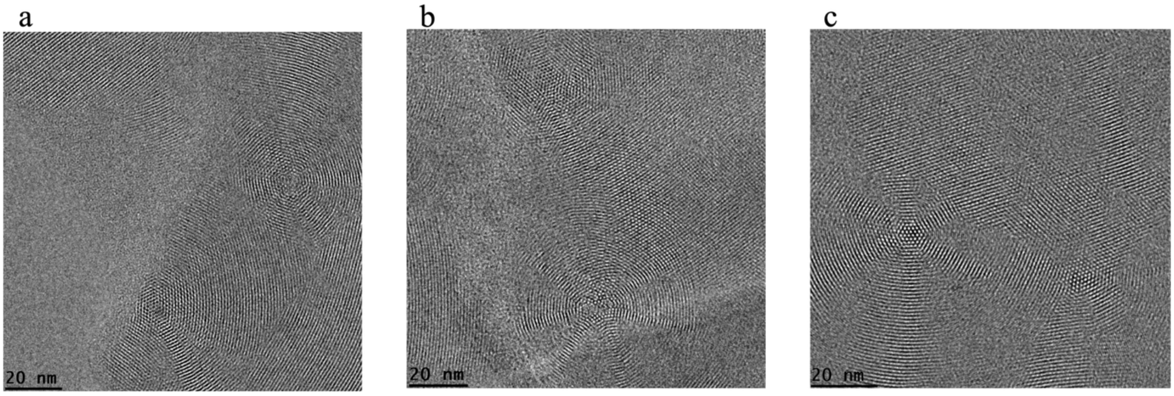

Figure S1. Additional High Resolution TEM images of different NaPHI flakes. Line, multi-rotation moire patterns, and other features are observed, indicating variance in crystallinity.

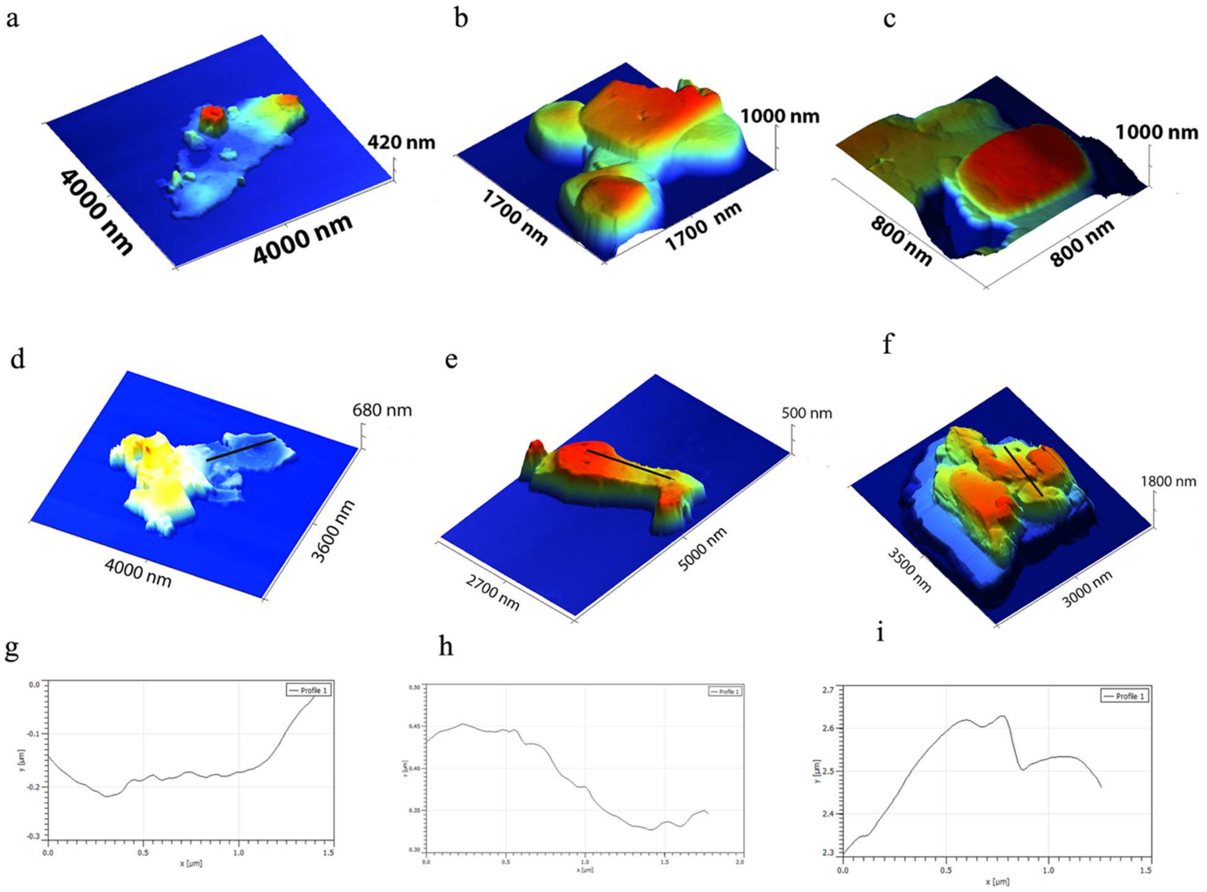

Figure S2. (a-f) Additional AFM images of NaPHI flakes. (g-i) line profiles from marked line in (d-f), respectively. Heterogeneity and curvature are observed.

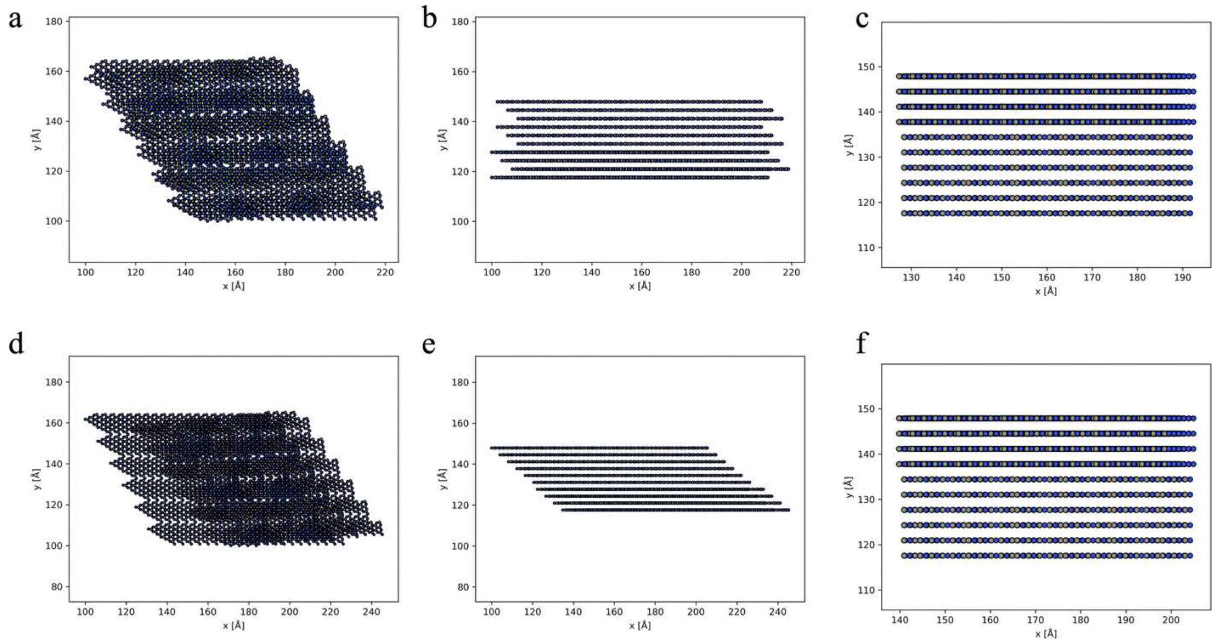

Figure S3. NaPHI stacking faults. (a-c) 10 layers of NaPHI with an abc stacking fault. (d-f) N10 layers of NaPHI with a continus stacking. Stacking distance is 1/3 a unit cell. a,d top views. (b,e) side view showing faults (rotation around x axis), (c,f) side view perpendicular to (b,e) showing unaffected direction (rotation around y axis).

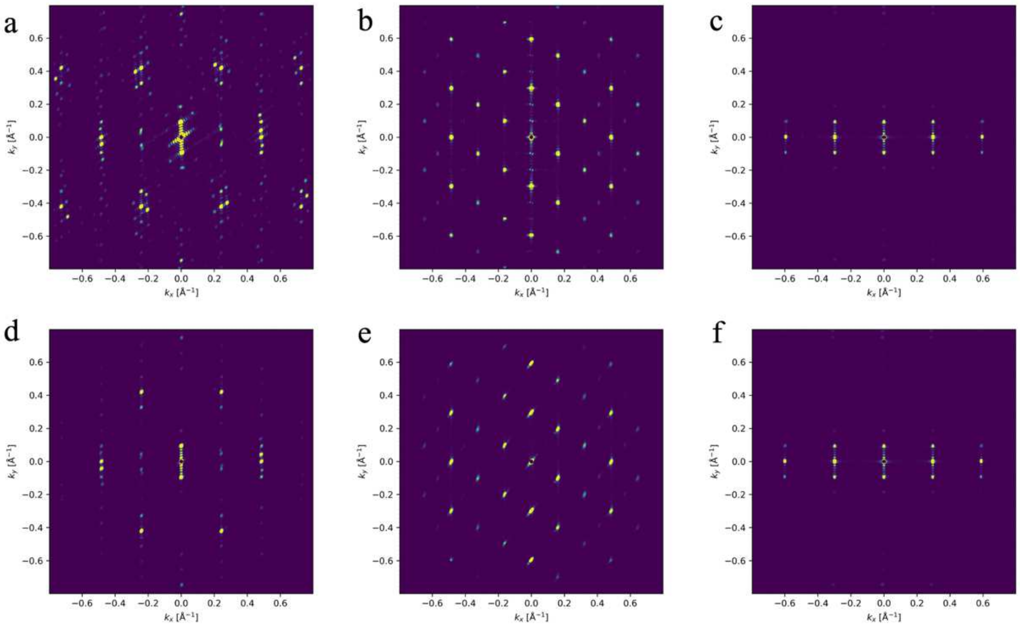

Figure S4. Simulated diffraction from stacking faults presented in Figure S3.

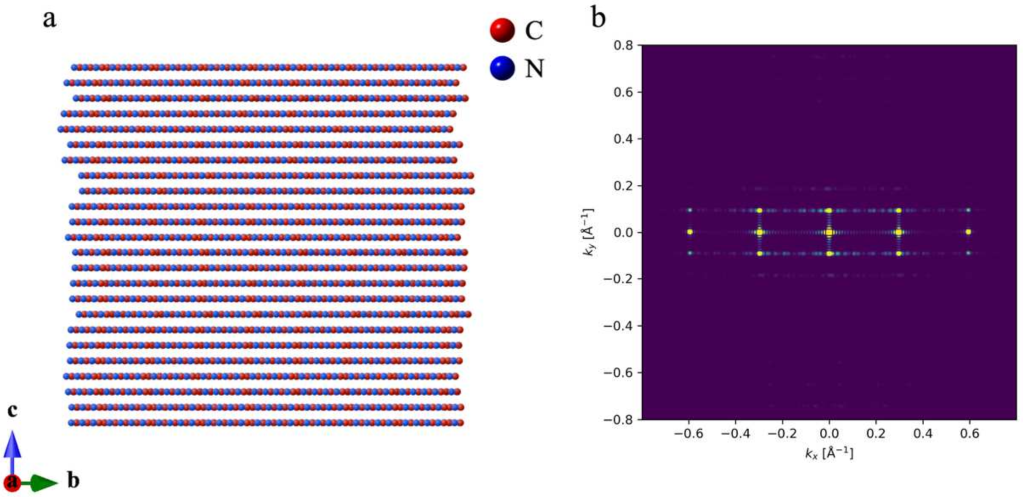

Figure S5 Small random shifts for stacking faults. (a) structure. (b) simulated diffraction. Line features are perpendicular to the direction seen in the data.

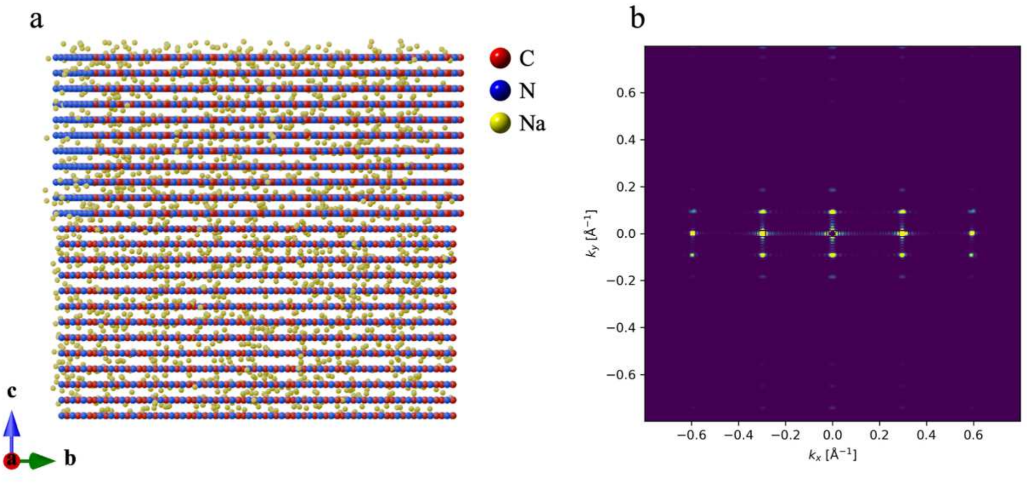

Figure S6 Testing the effect of random distribution of sodium ions across the sample. (a) structural model of NaPHI (8x8x8 nm) with sodium ions occupying 50% of active sites (approx. 1.5 ions per channel on average[10]). The ions are given random movements of 1.8Å in the channel (c axis) and 2Å in plane. Other values did not cause a meaningful change, and this structure was chosen for example. (b) simulated diffraction from the model in a. No line features are visible. Code is available in Git[7].

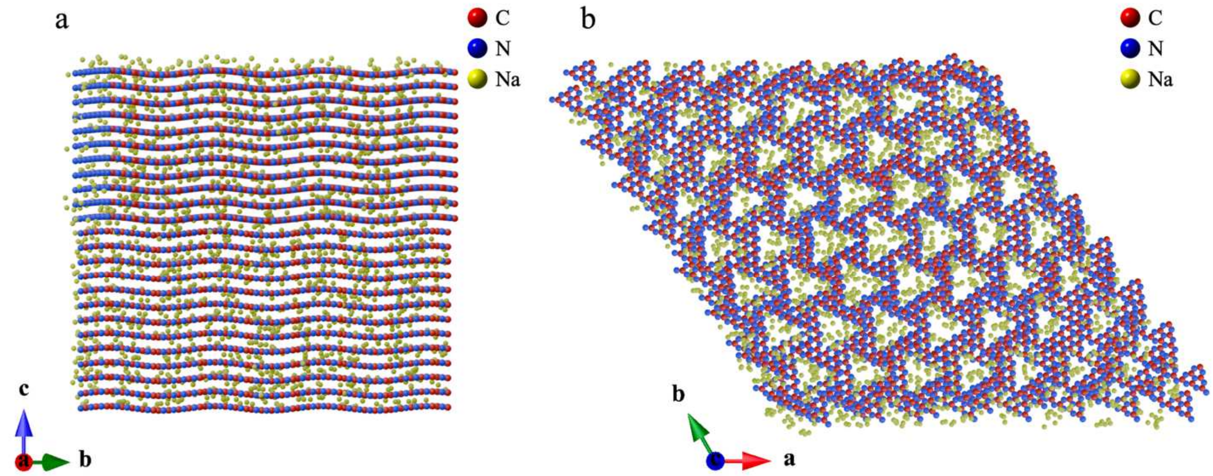

Figure S7 Structural model of NaPHI (8x8x8 nm), corresponding to structure in Figure 5a,d, with sodium ions occupying 50% of active sites (approx. 1.5 ions per channel on average[10]). The ions are given random movements of 1.8Å in the channel (c axis) and 2Å in plane. (a) side view. (b) top view.

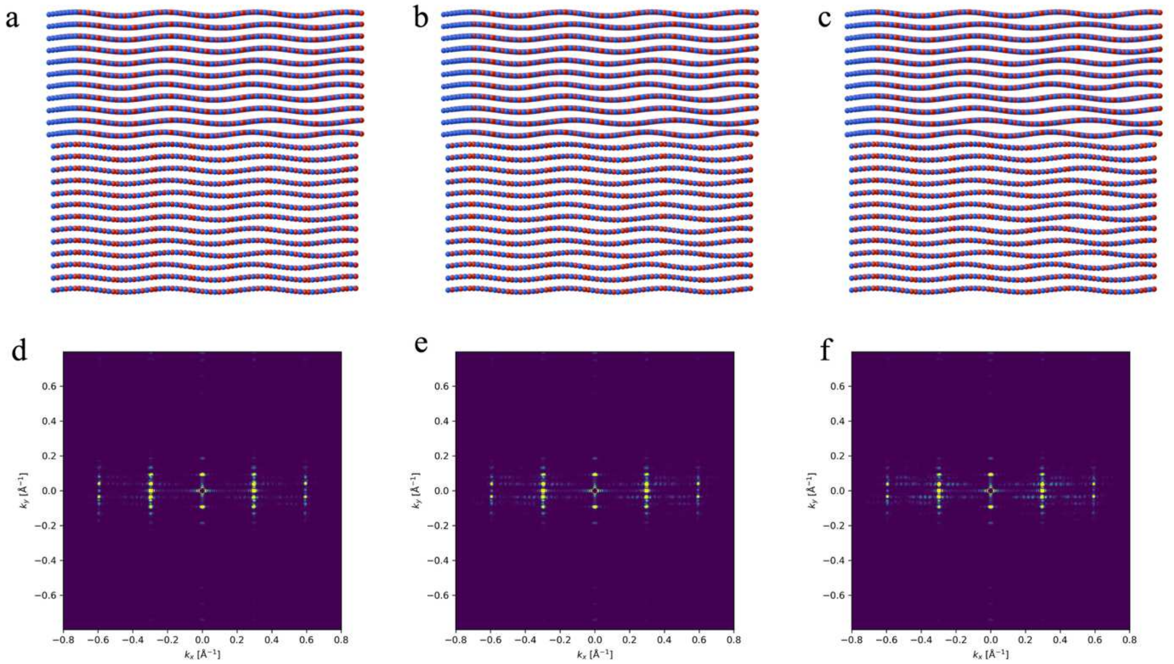

Figure S8 Variance in wavelengths and amplitudes for an initial wavelength of 24.8Å (unit cell x 2) and amplitude of 0.5Å. (a-c) 10%,15% and 20% variance in wavelengths and amplitudes, respectively. Each layer in the stack is given a base wavelength (24.8Å) and amplitude (0.5Å), and then each is multiplied by a random factor in a range of 0% to 10% (a), 15% (b) or 20% (c). Sodium ions are removed from the figure for viewing convenience. (d-f) simulated diffraction from structures a-c, respectively. For variance larger than 10% (b,e), small diffuse lines appear perpendicular to the central/line features, similarly to stacking-faults. While 15% may still be reasonable giving experimental conditions, at 10% they are not meaningful. For larger wavelengths, the diffuse lines appear at larger percentages.

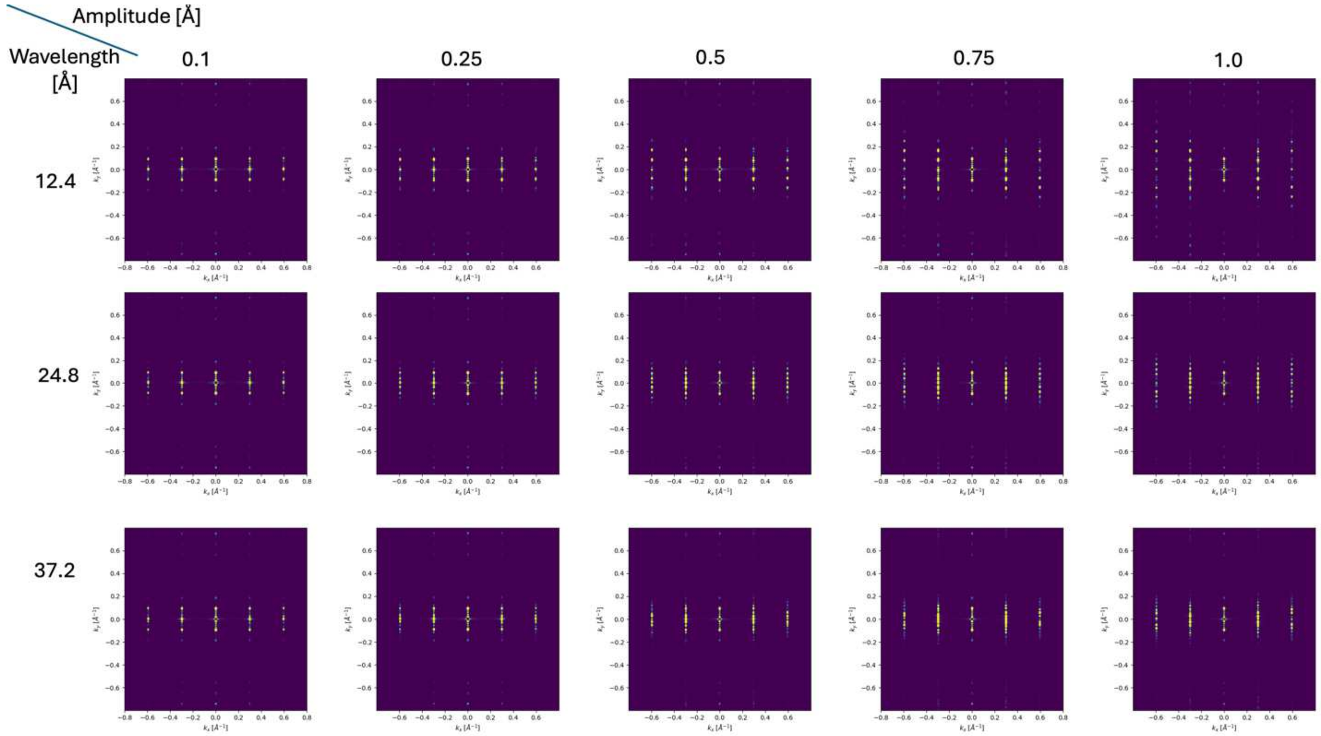

Figure S9. Effect of different wavelengths and amplitudes on abTEM simulations. Wavelengths were chosen as a full multiplicity of the unit cell. A minimum amplitude of 0.5Å and wavelength of 24.8Å are needed to form clear line features.

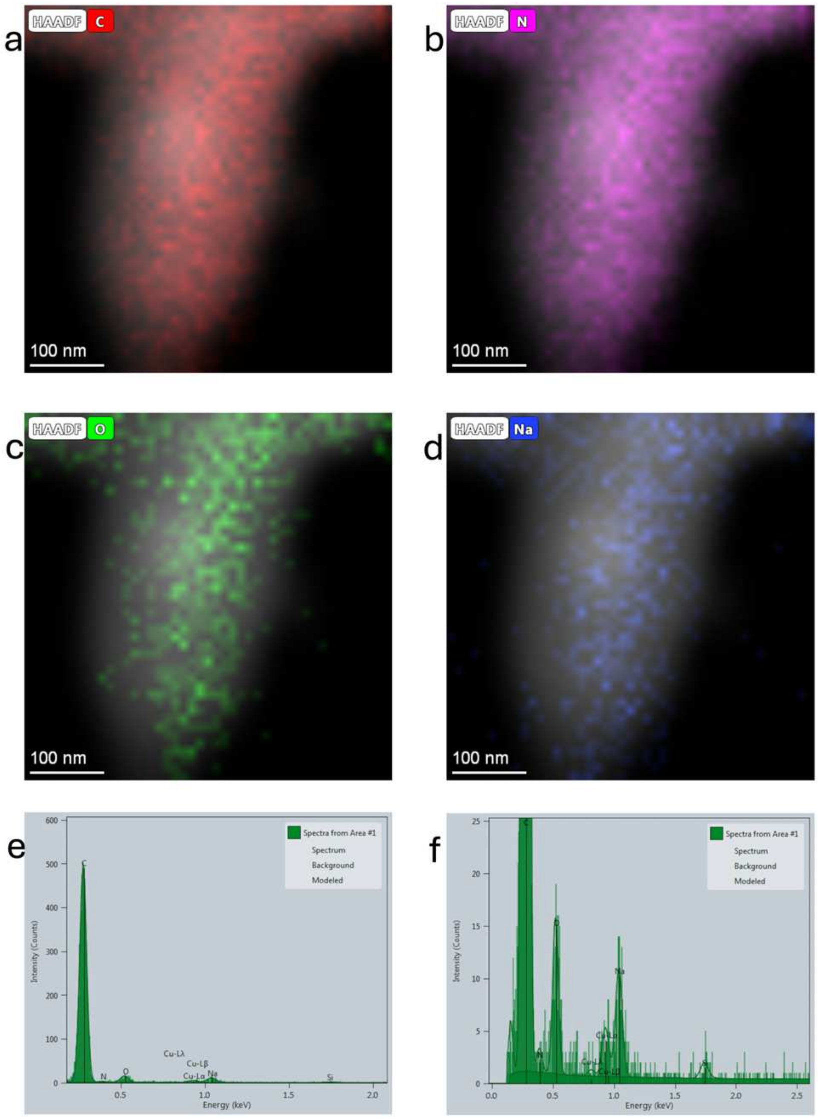

Figure S10. EDS of NaPHI. (a-d) elemental maps overlapped on HAADF for carbon, nitrogen oxygen and sodium, respectively. (e) EDS spectra and zoom-in including the background and fit. (f) zoomed-in spectra of e.

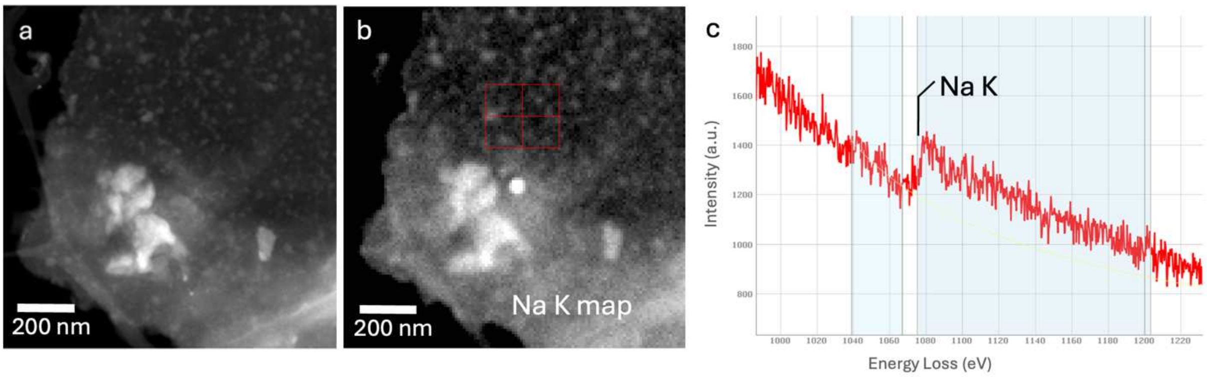

Figure S11. EELS Data. (a) HAADF image of collected area, (b) EELS map of Na edge at 1072 eV energy loss, (c) EELS spectrum from red box at b. The Na is, according to HAADF and EELS, not homogeneously incorporated.

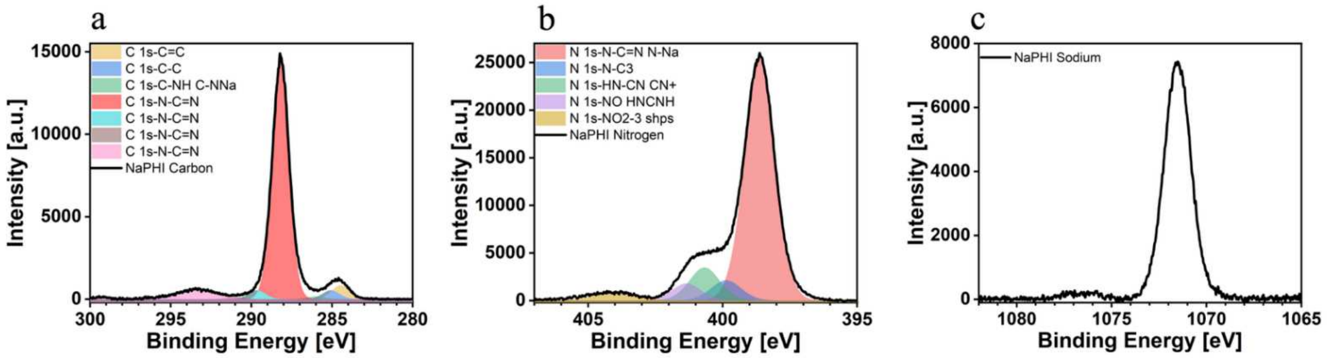

Figure S12. High resolution XPS spectra of NaPHI. (a) C 1s, (b) N 1s, and (c) Na 1s energy regions.

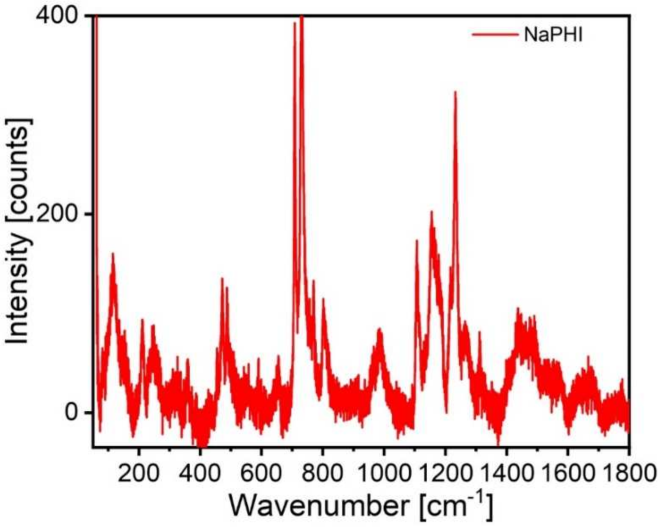

Figure S13. Raman spectrum of NaPHI. Characteristic heptazine breathing modes are observed at ~709 and ~982 cm^{-1}, confirming the presence of the heptazine framework. A weaker peak at ~753 cm^{-1} is assigned to out-of-plane bending of graphitic domains. These assignments are consistent with previous reports on PHI and related carbon nitride materials[14,15]

__References__

(1) Teixeira, I. F.; Tarakina, N. V.; Silva, I. F.; López-Salas, N.; Savateev, A.; Antonietti, M. Overcoming Electron Transfer Efficiency Bottlenecks for Hydrogen Production in Highly Crystalline Carbon Nitride-based Materials. *Adv. Sustain. Syst.* **2022**, *6* (3), 2100429.

(2) Nečas, D.; Klapetek, P. Gwyddion: An Open-Source Software for SPM Data Analysis. *Open Phys.* **2012**, *10* (1), 181–188.

(3) Kirillov, A.; Mintun, E.; Ravi, N.; Mao, H.; Rolland, C.; Gustafson, L.; Xiao, T.; Whitehead, S.; Berg, A. C.; Lo, W.-Y.; Dollár, P.; Girshick, R. Segment Anything. *arXiv [cs.CV]*, 2023. https://doi.org/10.48550/ARXIV.2304.02643.

(4) Bradski, G. R.; Pisarevsky, V. *Open Source Computer Vision Library*; Springer: New York, NY, 2004.

(5) *Matplotlib Is a 2D Graphics Package Used for Python for Application Development, Interactive Scripting, and Publication-Quality Image Generation across User Interfaces and Operating Systems.*

(6) van der Walt, S.; Schönberger, J. L.; Nunez-Iglesias, J.; Boulogne, F.; Warner, J. D.; Yager, N.; Gouillart, E.; Yu, T.; scikit-image contributors. Scikit-Image: Image Processing in Python. *PeerJ* **2014**, *2*, e453.

(7) Khaykelson, D. *NaPHI Repository*. GitHub. https://github.com/DanielKhaykelson/NaPHI_structural-simulations_SAM.

(8) Hjorth Larsen, A.; Jørgen Mortensen, J.; Blomqvist, J.; Castelli, I. E.; Christensen, R.; Dułak, M.; Friis, J.; Groves, M. N.; Hammer, B.; Hargus, C.; Hermes, E. D.; Jennings, P. C.; Bjerre Jensen, P.; Kermode, J.; Kitchin, J. R.; Leonhard Kolsbjerg, E.; Kubal, J.; Kaasbjerg, K.; Lysgaard, S.; Bergmann Maronsson, J.; Maxson, T.; Olsen, T.; Pastewka, L.; Peterson, A.; Rostgaard, C.; Schiøtz, J.; Schütt, O.; Strange, M.; Thygesen, K. S.; Vegge, T.; Vilhelmsen,

L.; Walter, M.; Zeng, Z.; Jacobsen, K. W. The Atomic Simulation Environment—a Python Library for Working with Atoms. *J. Phys. Condens. Matter* **2017**, *29* (27), 273002.

(9) Madsen, J.; Susi, T. The AbTEM Code: Transmission Electron Microscopy from First Principles. *Open Res. Eur.* **2021**, *1*, 24.

(10) Piankova, D. V.; Zschiesche, H.; Tyutyunnik, A. P.; Svensson Grape, E.; da Silva, C. V. C. R.; Guimarães Junior, W. G.; de Moura, A. F.; Reis, I. F.; Diab, G. A. A.; Filho, J. B. G.; Teixeira, I. F.; Tarakina, N. V. Enhancing the Photocatalytic Performance of Carbon Nitrides through Controlled Local Structure Modification. *Adv. Funct. Mater.* **2025**, e11389.

(11). https://github.com/DanielKhaykelson/NaPHI_structural-simulations_SAM/tree/main (Accessed 2025-07-02).

(12) Debye, P. Zerstreuung von Röntgenstrahlen. *Ann. Phys.* **1915**, *351* (6), 809–823.

(13) Houben, L.; Enyashin, A. N.; Feldman, Y.; Rosentsveig, R.; Stroppa, D. G.; Bar-Sadan, M. Diffraction from Disordered Stacking Sequences in MoS_2 and WS_2 Fullerenes and Nanotubes. *J. Phys. Chem. C* **2012**, *116* (45), 24350–24357.

(14) Miller, T. S.; Jorge, A. B.; Suter, T. M.; Sella, A.; Corà, F.; McMillan, P. F. Carbon Nitrides: Synthesis and Characterization of a New Class of Functional Materials. *Phys. Chem. Chem. Phys.* **2017**, *19* (24), 15613–15638.

(15) Rogolino, A.; Silva, I. F.; Tarakina, N. V.; da Silva, M. A. R.; Rocha, G. F. S. R.; Antonietti, M.; Teixeira, I. F. Modified Poly(Heptazine Imides): Minimizing H2O2 Decomposition to Maximize Oxygen Reduction. *ACS Appl. Mater. Interfaces* **2022**, *14 (44)*, 49820—49829.
